# Determination of the Potential Clinical Benefits of Small Molecule Factor XIa Inhibitors in Arterial Thrombosis

**DOI:** 10.1021/acsptsci.3c00052

**Published:** 2023-06-30

**Authors:** Surasak Wichaiyo, Warisara Parichatikanond, Satsawat Visansirikul, Nakkawee Saengklub, Wipharak Rattanavipanon

**Affiliations:** † Department of Pharmacology, Faculty of Pharmacy, Mahidol University, Bangkok 10400, Thailand; ‡ Centre of Biopharmaceutical Science for Healthy Ageing, Faculty of Pharmacy, Mahidol University, Bangkok 10400, Thailand; § Department of Pharmaceutical Chemistry, Faculty of Pharmacy, Mahidol University, Bangkok 10400, Thailand; ∥ Department of Physiology, Faculty of Pharmacy, Mahidol University, Bangkok 10400, Thailand; ⊥ Department of Pharmacy, Faculty of Pharmacy, Mahidol University, Bangkok 10400, Thailand

**Keywords:** activated factor XI, FXIa, small molecule FXIa inhibitors, arterial thrombosis, ischemic stroke, myocardial infarction

## Abstract

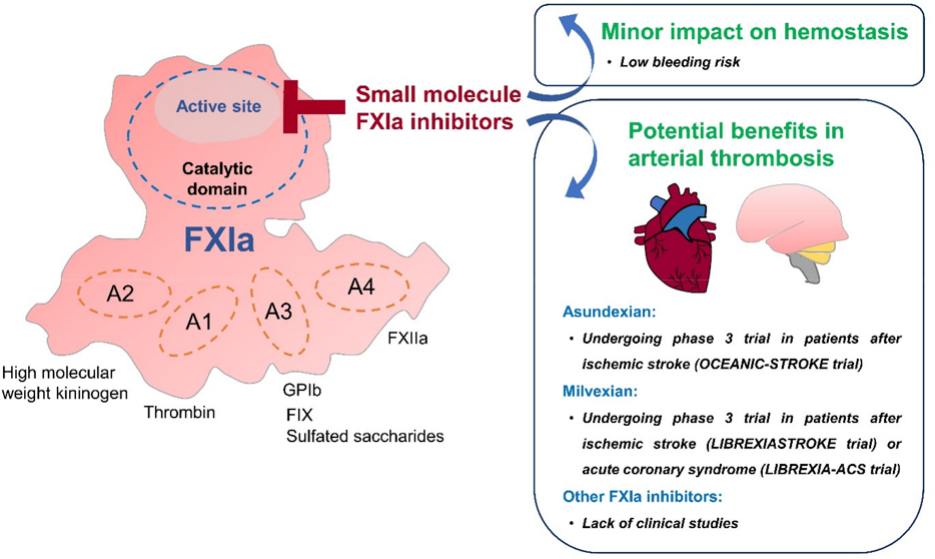

Anticoagulants are the mainstay for the prevention and treatment of thrombosis. However, bleeding complications remain a primary concern. Recent advances in understanding the contribution of activated factor XI (FXIa) in arterial thrombosis with a limited impact on hemostasis have led to the development of several FXIa-targeting modalities. Injectable agents including monoclonal antibodies and antisense oligonucleotides against FXIa have been primarily studied in venous thrombosis. The orally active small molecules that specifically inhibit the active site of FXIa are currently being investigated for their antithrombotic activity in both arteries and veins. This review focuses on a discussion of the potential clinical benefits of small molecule FXIa inhibitors, mainly asundexian and milvexian, in arterial thrombosis based on their pharmacological profiles and the compelling results of phase 2 clinical studies. The preclinical and epidemiological basis for the impact of FXIa in hemostasis and arterial thrombosis is also addressed. In recent clinical study results, asundexian appears to reduce ischemic events in patients with myocardial infarction and minor-to-moderate stroke, whereas milvexian possibly provides benefits in patients with minor stroke or high-risk transient ischemic attack (TIA). In addition, asundexian and milvexian had a minor impact on hemostasis even in combination with dual-antiplatelet therapy. Other orally active FXIa inhibitors also produce antithrombotic activity in vivo with low bleeding risk. Therefore, FXIa inhibitors might represent a new class of direct-acting oral anticoagulants (DOACs) for the treatment of thrombosis, although the explicit clinical positions of asundexian and milvexian in patients with ischemic stroke, high-risk TIA, and coronary artery disease require confirmation from the outcomes of ongoing phase 3 trials.

Anticoagulants play an important role in the prevention and treatment of cardioembolism^1^ and venous thromboembolism (VTE).^2,3^ In addition, rapid-acting anticoagulants, such as heparin and its derivatives, might be used in combination with antiplatelets in acute settings during arterial thrombosis, including in patients with acute coronary syndrome undergoing percutaneous coronary intervention.^4,5^ Although several classes of anticoagulant are available to date, bleeding complications remain a major concern. Heparin^2,6^ and warfarin^1^ have a narrow therapeutic window, which requires frequent therapeutic monitoring to prevent treatment failure (e.g., recurrent thrombosis) or overdose (e.g., adverse bleeding events). Direct-acting oral anticoagulants (DOACs) are relatively preferable given that a specific laboratory test is not required for their use.^2,7^ However, major bleeding and potential drug interactions are reported in clinical practice.^2,7^ Therefore, novel targets that act as a key regulator in thrombosis with a limited impact on hemostasis are of particular interest for the discovery and development of new anticoagulants.

Recently, epidemiological data have shown that factor XI (FXI) plays an important role in thrombosis in both arteries and veins.^8^ High levels of FXI are associated with VTE events, and patients with FXI deficiency are protected from VTE.^8^ Therefore, various FXI-targeting approaches, including monoclonal antibodies (e.g., abelacimab, osocimab, and xisomab 3G3), aptamers (e.g., 11.16 and 12.7), antisense oligonucleotides (e.g., IONIS-FXIRx), and small molecules, have been investigated for the prevention and treatment of VTE.^9−11^ Most of these novel drug candidates have succeeded in phase 2 clinical trials.^9,10,12^ The aptamers are in preclinical investigation.^9,10,12^ A meta-analysis of phase 2 studies in patients undergoing total knee arthroplasty reported that FXI-targeting agents were more effective in VTE prevention, with a lower bleeding risk, than low molecular weight heparins.^9^

At present, the evidence has suggested that increased levels of activated FXI (FXIa) contribute to the risk of arterial thrombosis, including ischemic stroke and myocardial infarction.^8^ While the current development of monoclonal antibodies and antisense oligonucleotides against FXIa is mainly directed toward the prevention and treatment of VTE,^9,10^ preclinical and clinical studies have reported the potential therapeutic role of small molecule FXIa inhibitors in both venous and arterial thrombosis settings.^10,13^ In addition, many small molecule FXIa inhibitors are orally active, providing advantages over monoclonal antibodies, antisense oligonucleotides, and aptamers, which require a parenteral route of administration.^11^ In agreement with this, a survey study in cancer patients who experienced thromboembolic complications and care givers reported that a safe and effective oral anticoagulant is preferable to an injectable formulation.^14,15^ A simplified drug regimen is also one of the methods to improve medication adherence for the treatment and prevention of arterial thrombotic diseases given that good adherence to drug therapy is crucial for clinical outcomes.^16^ A meta-analysis evaluating the association between medication adherence and mortality has suggested that patients with good medication adherence, including postmyocardial infarction, were associated with a reduction in mortality of approximately 50% compared to those with poor adherence.^17^ Therefore, orally active small molecule FXIa inhibitors could potentially be an interesting option for the management of arterial thrombosis. This review summarizes the impact of FXI in arterial thromboinflammation, demonstrates the potential binding sites of small molecules targeting FXIa, and describes the pharmacological properties of FXIa inhibitors, particularly asundexian and milvexian, which have recently completed phase 2 clinical studies in patients after recent ischemic stroke or myocardial infarction.

## Role of Factor XI (FXI) in Thrombosis and Hemostasis

**Figure 1:**
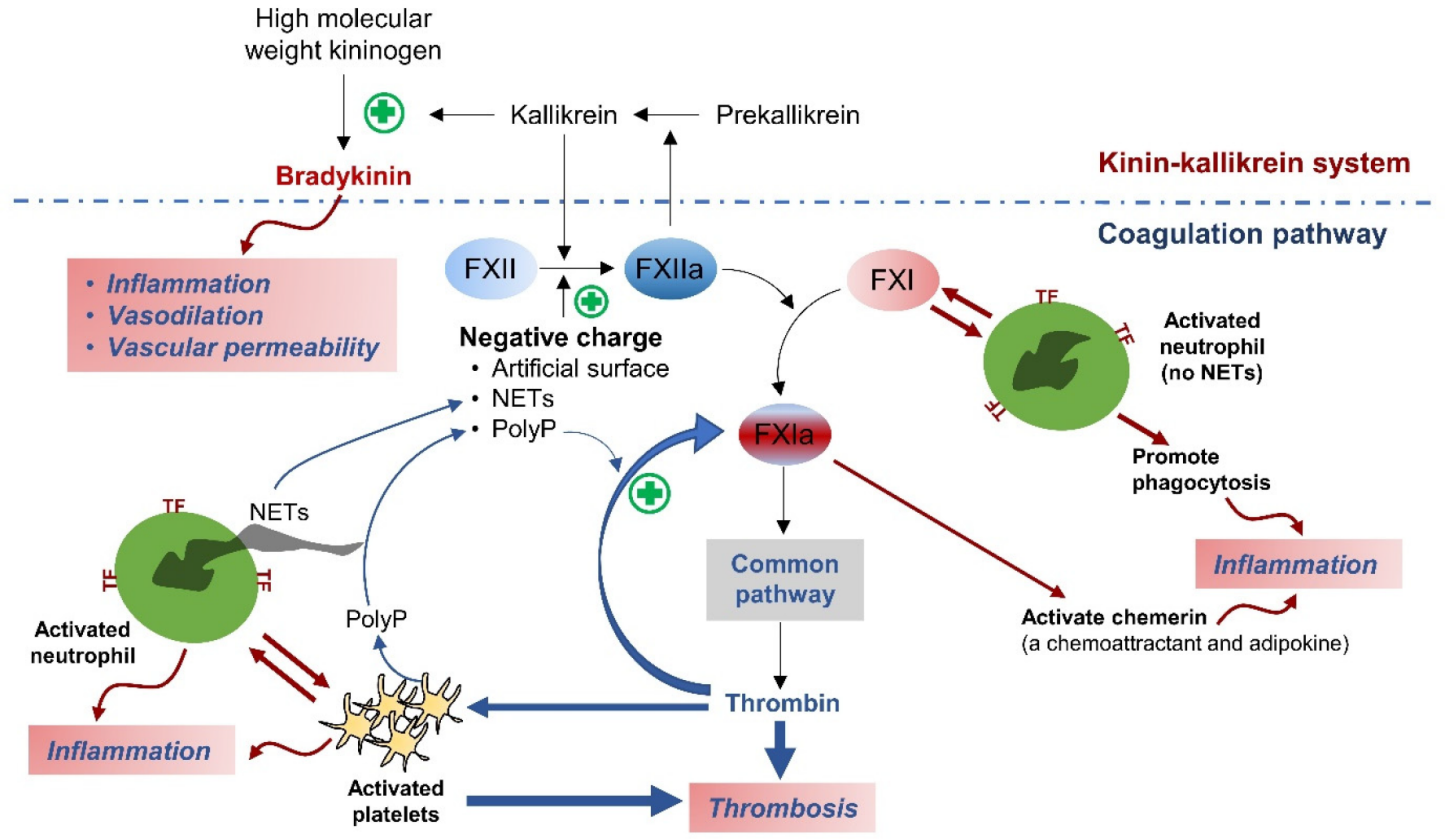
Cross talk between coagulation, platelets, and inflammation. The intrinsic (contact) pathway of coagulation is initiated by the activation of FXII by negatively charged molecules such as polyphosphate (PolyP) from activated platelets, DNA from neutrophil extracellular traps (NETs), and artificial devices. The activated FXII (FXIIa) then stimulates FXI to FXIa and leads to thrombin generation. Thrombin and activated factor X (FXa) in the common pathway are capable of stimulating platelets, which further promote thrombus formation. PolyP secreted from activated platelets in turn amplifies thrombin-mediated FXI activation. In addition, activated platelets interact with neutrophils, which leads to neutrophil activation. Tissue factor (TF) expressed on activated neutrophils might trigger the extrinsic pathway of coagulation. The activated neutrophils also directly activate FXI, whereas FXI promotes the phagocytic activity of neutrophils. Furthermore, FXIa contributes to the activation of chemerin, a chemoattractant, and adipokine that promotes leukocyte migration to the inflammatory site. Apart from that, the intrinsic pathway is closely linked to the kinin–kallikrein system. FXII is activated to FXIIa by kallikrein. Simultaneously, FXIIa converts prekallikrein to active kallikrein and generates bradykinin, which contributes to inflammation.

Extrinsic and intrinsic pathways of coagulation play a role in clot formation.^18,19^ Following endothelial injury, such as atherosclerotic plaque rupture, tissue factor (TF) at the perivascular area promotes the initiation of clot formation, contributing to atherothrombosis.^18,20−23^ In addition, the extrinsic (TF-activated factor VII) pathway is activated in the absence of vascular injury given that TF is expressed on monocytes, macrophages, and neutrophils following inflammation, which could be seen in venous thrombosis.^19,20,24−26^ Despite this, thrombin generation via the extrinsic pathway is minimal given that it is inhibited by tissue factor pathway inhibitor (TFPI).^19^ The intrinsic pathway, which comprises FXI, has been shown to play an important role in sustaining thrombin generation (Figure 1). Therefore, FXI potentially contributes to thrombus propagation and stabilization.^19,22,23,27^

In human plasma, the FXI levels range between 3 and 7 μg/mL and the normal FXI coagulant activity (FXI:C) is approximately 70–150 U/dL.^19^ Generally, FXI complexes with high molecular weight kininogen to maintain its stability in circulation (half-life ≈ 52 h) and facilitate binding to negatively charged molecules that promote its activation to FXIa.^19^ Although the intrinsic pathway amplifies clot propagation, it is less likely that FXI contributes to hemostasis.^18,19^ In patients with FXI deficiency, it has been reported that spontaneous bleeding is rarely observed, but the bleeding risk is increased following injuries and surgeries.^28−33^ More recently, a large retrospective cohort study confirmed that FXI deficiency (FXI activity < 50%) was associated with an increased risk of severe bleeding and clinically relevant nonsevere bleeding, primarily postprocedure.^34^ This phenotype is unlike the more frequent spontaneous bleeding found in patients with hemophilia A (factor VIII deficiency) and hemophilia B (factor IX deficiency),^28,35^ supporting a minor role of FXI in hemostasis.

## FXI and the Link between Thrombosis and Inflammation

Coagulation, platelet activation, and inflammation are interrelated. In addition to their role in promoting fibrin formation,^36^ thrombin and activated factor X (FXa) are capable of stimulating platelets by activating the protease-activated receptors (Figure 1).^37,38^ Given that FXI is an upstream factor of FXa and thrombin, it might be possible that FXI contributes to platelet function.^39^ In addition, it has been shown that FXI directly binds to glycoprotein Ibα and apolipoprotein E receptor 2 on the platelet surface, resulting in platelet activation.^40,41^ Activated platelets secrete polyphosphate anions to promote FXIIa generation and amplify thrombin-mediated FXI activation in the intrinsic (contact) pathway (Figure 1).^42,43^ Moreover, interaction between activated platelets and neutrophils stimulates a release of neutrophil extracellular traps (NETs) to promote inflammation.^44^ NETs contain DNA (a negatively charged molecule), which leads to FXII activation and additional thrombin generation (Figure 1).^45,46^ In addition, it has been reported that the activated neutrophil itself may promote clotting in a FXI-dependent manner.^47^ Moreover, the contact pathway contributes to the synthesis of bradykinin, a potent proinflammatory mediator, in the kinin–kallikrein system (Figure 1). FXIIa converts prekallikrein to active kallikrein, which cleaves high molecular weight kininogen and generates bradykinin.^46,48^ Kallikrein in turn promotes coagulation by activating FXII. Together, this evidence indicates the crosstalk between inflammation and thromboembolism.

Currently, it is unclear whether FXI directly mediates inflammatory responses. At least, FXI might promote the phagocytic activity of neutrophils (Figure 1) following inflammation such as sepsis induced by *Streptococcus pneumoniae* or *Klebsiella pneumoniae* pneumonia.^49^ In plasma, it has been shown that FXIa cleaves prochemerin to an intermediate that could be further processed to active chemerin (Figure 1), a chemoattractant, and adipokine.^50^ In addition, serum chemerin levels were increased in patients with acute ischemic stroke and carotid artery atherosclerosis.^51^ These observations potentially link FXI-mediated inflammation in atherosclerosis and arterial thrombosis.

## Atherosclerosis and Arterial Thrombosis in FXI-Deficient Animals

**Figure 2:**
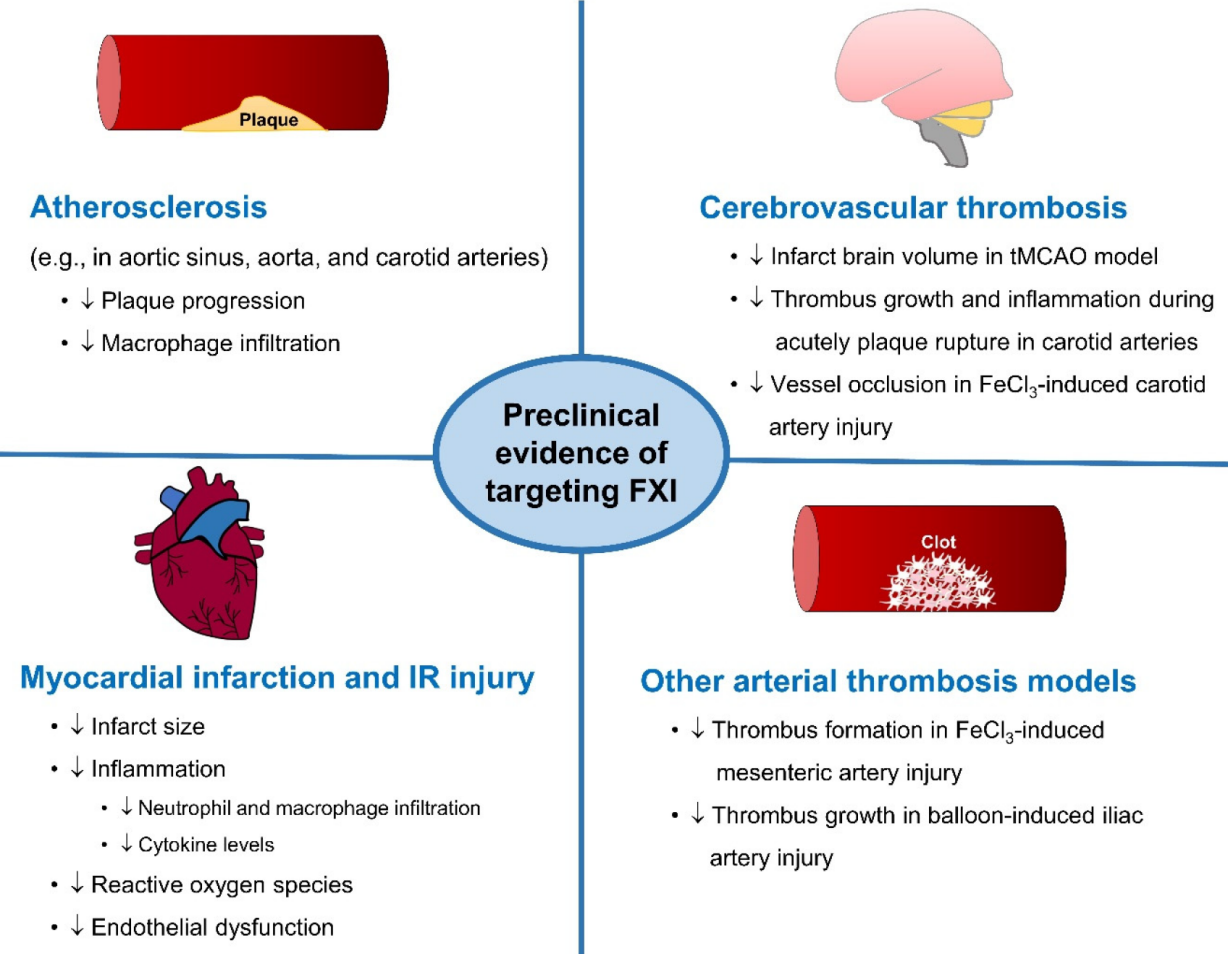
Preclinical evidence for the potential impact of targeting factor XI (FXI) in atherosclerosis and arterial thrombosis. tMCAO = transient middle cerebral artery occlusion. ↓ = decrease. A reduction in FXI activity attenuates the progression of atherosclerotic plaque and thrombus growth in cardio- and cerebrovascular thrombosis. In addition, targeting FXI reduces inflammation (e.g., decreased infiltration of neutrophils and macrophages and/or cytokine levels) during atherosclerosis, myocardial ischemia–reperfusion (IR) injury, and carotid arterial thrombosis. Moreover, the potential antithrombotic activity of inhibiting FXI is reported in other models of arterial thrombosis, including ferric chloride (FeCl_3_)-induced mesenteric artery injury and balloon-induced iliac artery injury.

Several lines of evidence have demonstrated the roles of FXI in atherosclerosis and arterial thrombosis. In a model of atherosclerosis (apolipoprotein E knockout, *Apoe*^*–/–*^), mice with a double knockout of FXI and APOE (*F11*^*–/–*^, *Apoe*^*–/–*^) had an attenuated progression of atherosclerosis with reduced macrophage infiltration in atherosclerotic lesions on the aortic sinus and aortic arch compared to *Apoe*^*–/–*^ alone (Figure 2).^52^ In mice deficient in low-density lipoprotein receptor (*Ldlr*^*–/–*^) fed high-fat diets for 8–16 weeks, the 14E11 antibody, which inhibited the activation of FXI by FXIIa, reduced atherosclerotic lesion in the proximal aorta.^53^ In addition, targeting FXI using antisense oligonucleotide (FXI-ASO) inhibited atherosclerosis progression following high-fat diet treatment in *Ldlr*^*–/–*^ mice.^53^

During a model of myocardial ischemia–reperfusion injury in mice, the 14E11 antibody significantly reduced the infarct size (Figure 2).^54^ Moreover, it has been reported that FXI-ASO attenuates inflammation in mice with myocardial ischemia–reperfusion injury,^55^ indicated by the decreased influx of neutrophils and monocytes to the ischemic myocardium, in association with a reduction in endothelial dysfunction, reactive oxygen species (ROS) production, and cytokine levels, including interleukin (IL)-6, IL-1, and macrophage-1 antigen (MAC-1) (Figure 2).^55^

The role of FXI has also been investigated in cerebrovascular thrombosis. The infract brain volume and intravascular fibrin content were decreased in FXI null mice during transient middle cerebral artery occlusion (tMCAO), an acute ischemic stroke model (Figure 2).^56^ In ferric chloride (FeCl_3_)-induced carotid artery injury, *F11*^*–/–*^ mice were protected against vessel occlusion, with no alteration in bleeding time, compared to a complete block of carotid artery blood flow in the control animals.^57^ In addition, FXI-ASO reduced thrombus formation and inflammation (infiltration of macrophages) in acutely ruptured atherosclerotic plaque in the carotid arteries of *Apoe*^*–/–*^ mice fed with a high-fat diet (Figure 2).^58^ Moreover, a reduction in thrombus formation due to FXIa deficiency was observed in other arterial thrombosis models, including FeCl_3_-induced injury of mesenteric arterioles in mice^59^ and balloon-induced injury of the neointima of the iliac artery in rabbits, without affecting bleeding time.^60^

Together, these studies demonstrate a significant contribution of FXI in arterial thromboinflammation, including atherosclerotic plaque progression, myocardial infarction, and cerebrovascular thrombosis. Therefore, targeting FXI may provide benefits in atherosclerosis and arterial thrombosis with a potentially low bleeding risk.

## FXI and Risk of Arterial Thrombosis in Humans

Several studies have reported that FXI is associated with an increased risk of arterial thrombosis.^8^ A retrospective study in 65 stroke patients suggested that high FXI activity was associated with an increased risk of stroke.^61^ This observation was confirmed by a prospective cohort study in 621 mild-to-moderate ischemic stroke patients that revealed a strong association between high FXI activity and secondary stroke event following a 3-year follow up.^62^ In young women (18–50 year old) who had cardiovascular risk factors such as smoking, hypertension, dyslipidemia, and diabetes mellitus, it has been shown that increased levels of FXIa were associated with ischemic stroke.^63^ In addition, oral contraceptive use potentiated the risk of ischemic stroke.^63^ However, a study in the general population with no history of stroke and coronary heart disease reported that high plasma FXI levels did not correlate with the incidence of stroke.^64^ Notably, there is a lack of apparent data in these studies to indicate whether the large vessel atherosclerosis or cardioembolic origin is primarily affected by alterations in FXI activity.

On the contrary, a study in Israel showed that individuals with mild FXI deficiency (FXI activity 30–50%) or moderate-to-severe FXI deficiency (FXI activity ≤ 30%) had a lower incidence of composite cardiovascular events, including myocardial infarction, stroke, and transient ischemic attack (TIA).^65^ In addition, 115 patients aged over 45 years who had severe FXI deficiency (FXI activity < 15 U/dL) demonstrated a substantial reduction in incidence of ischemic stroke relative to the expected cases in the general population.^66^ This evidence suggests that FXI is a potential target of novel antithrombotic agents for the treatment of ischemic stroke.

It has been revealed that FXIa levels are elevated during the acute phase of myocardial infarction, independent of FXIIa.^67^ In agreement with this, plasma levels of FXIa exhibited a positive relationship with cardiovascular events in patients with stable coronary artery disease.^68^ Moreover, a sex difference was observed in the relationships between FXI levels and the risk of myocardial infarction. In men who had cardiovascular risk factors, an elevated FXI level has been demonstrated to increase the risk of myocardial infarction.^69^ This correlation was not observed in women with those risk factors,^63,70^ suggesting that males might be more reactive to FXI. Similarly, in the absence of an apparent association between myocardial infarction and severe FXI deficiency among 115 Israeli patients, FXI-deficient males appeared to show higher numbers of affected myocardial infarction (14 cases in a total of 53 men; 26%) than females (5 cases in a total of 62 women; 8%).^66^ Further studies are required to confirm this observation of sex difference. Again, high plasma FXI levels in the general population were not associated with the incidence of coronary heart disease.^64^

## Target Sites of Small Molecule Activated Factor XI (FXIa) Inhibitors

**Figure 3:**
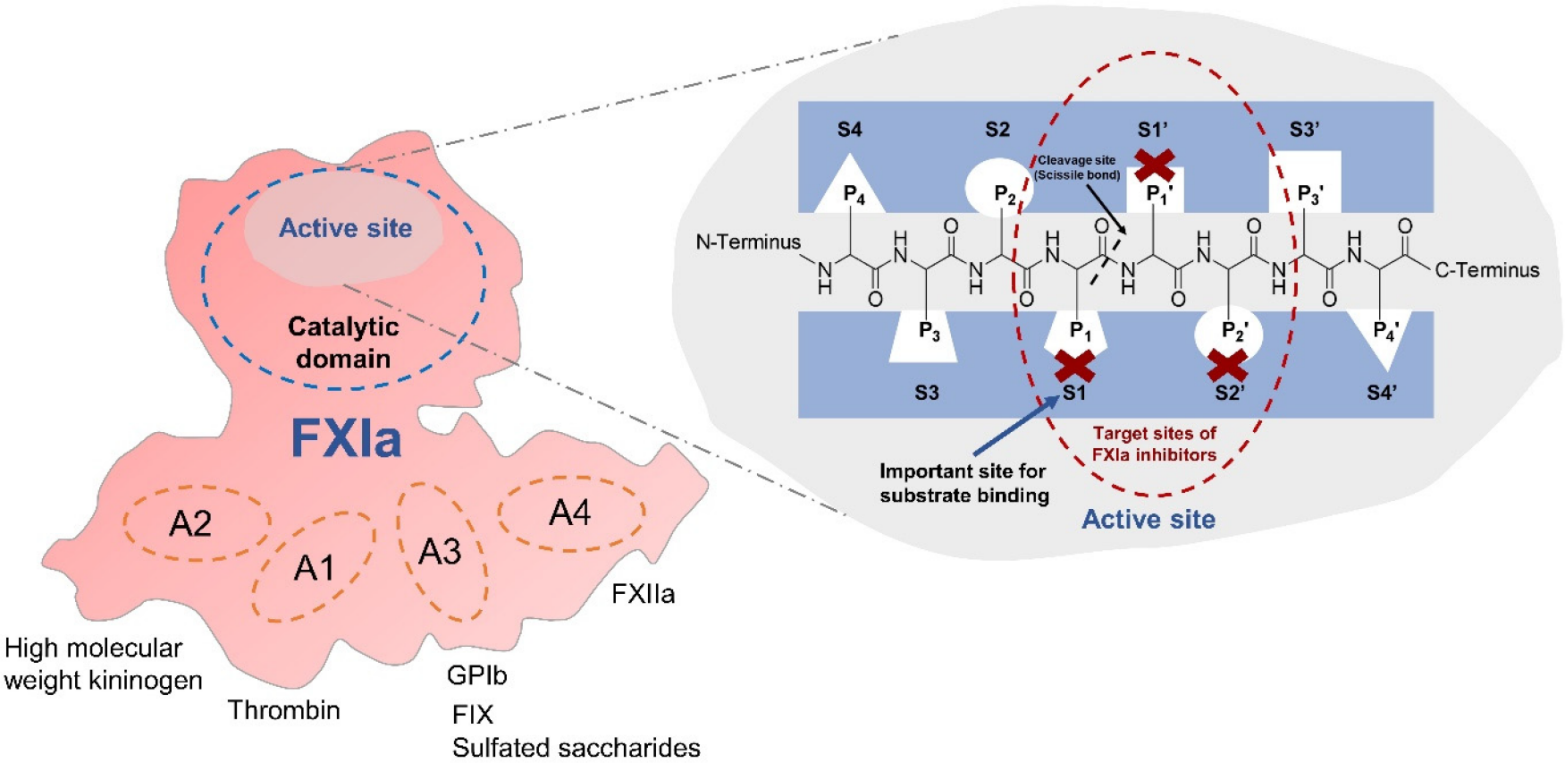
Structural domains of activated factor XI (FXIa) and target sites of FXIa inhibitors. Human FXIa is a homodimeric serine protease. One unit of FXI comprises a single catalytic domain and four apple (A) domains. A1–A4 are responsible for interacting with macromolecules. The active site in the catalytic domain includes eight subsites (i.e., S1–S4 and S1′–S4′). The S1 subsite is highly important for substrate binding. S1, S1′, and S2′ represent the potential target sites of FXIa inhibitors. GPIb = glycoprotein Ib, FIX = factor IX, FXIIa = activated factor XII.

Human FXIa is a serine protease, which is comprised of two units linked by disulfide bonds. Each unit has five domains: one catalytic domain and four apple (A) domains (Figure 3).^71^ Interacting macromolecules bind in the A domains, including high molecular weight kininogen in the A1/A2 domains, thrombin in the A1 domain, platelet glycoprotein Ib, clotting factor IX, and sulfated saccharides in the A3 domain, and FXIIa in the A4 domain.^71^ The active site in the catalytic domain has eight subsites (Figure 3). The S1′, S2′, S3′, and S4′ subsites correspond to the C-terminus side of the cleavage bond, whereas the S1, S2, S3, and S4 subsites represent the N-terminus side of the cleavage bond.^71^ The important subsite highly responsible for substrate binding of human FXIa is S1.^71^

At present, several small molecules have been developed as FXIa inhibitors, but their specificity is a concern given that the active site of FXIa is relatively similar to other serine proteases.^72^ Generally, the small molecules were aimed to specifically bind to the S1, S1′, and S2′ subsites of FXIa given that they are the essential sites for the activation of the substrates (Figure 3).^71,73^ Among these small molecules, asundexian (a 2-oxopyridine-containing molecule) and milvexian (a macrocyclic derivative)^73,74^ represent specific FXIa inhibitors, which have recently demonstrated potential benefits in attenuating arterial thrombosis.

## Asundexian

Asundexian (BAY2433334) is a potent and specific FXIa inhibitor (Table 1).^75^ It binds to the active site of FXIa in a reversible, concentration-dependent manner.^75^ In human plasma, asundexian prolonged the activated partial thromboplastin time (aPTT), a test of the intrinsic pathway, with little effect on prothrombin time (PT), a test of the integrity of the extrinsic pathway.^75^ Asundexian demonstrated in vivo anticoagulant activity with a low bleeding risk, compared to many DOACs.^75^ In a rabbit model of FeCl_3_-induced damage to the carotid artery, asundexian exhibited a dose-dependent reduction in the thrombus weight without affecting the bleeding time, whereas rivaroxaban significantly prolonged the bleeding time. In combination with dual-antiplatelet therapy (aspirin and ticagrelor), asundexian produced antithrombotic activity with no increased risk of bleeding relative to an increase in bleeding time in apixaban-treated rabbits.^75^ Moreover, asundexian showed no impact on hemostasis in a rabbit model of liver injury, whereas dabigatran increased the bleeding time.^75^

**Table 1:** Pharmacodynamic/Pharmacokinetic Properties and Status of Development of Small Molecule Activated Factor XI (FXIa) Inhibitors^a^

parameters	asundexian^71,73,85^	milvexian^70,77,78,80,81^	SHR2285^81^	ONO-7684^82−84^	BMS-962212^85^	BMS-724296^86^	BMS-654457^87^	BMS-262084^88,89^
FXIa binding affinity (*K*_i_, nM)	1.0	0.1	NA	2.0	0.7	0.3	0.4	NA
oral bioavailability (%)	95 (tablet)	32^b^	NA	81^b^	NA (injectable)	NA	NA	NA
*T*_max_ (h)	1 (solution), 3–4 (tablet)	3–4 (tablet)	3–4 (tablet)	2.5–4 (tablet)	1–2	NA	NA	NA
plasma *t*_1/2_ (h)	14–17	8–14	8–16	16–20 (fasted), 22–28 (fed)	2–5	NA	NA	NA
metabolism	possibly CYP3A4	CYP3A4	NA	NA	NA	NA	NA	NA
current status of development	ongoing phase 3 minor stroke and high-risk TIA, AF	ongoing phase 3 minor stroke and high-risk TIA, ACS, AF	ongoing phase 2 TKR	completed phase 1	completed phase 1	preclinical carotid arterial thrombosis	preclinical carotid arterial thrombosis	preclinical carotid arterial thrombosis and venous thrombosis

a *K*_i_ = inhibition constant, NA = data is not available.

b Data in monkeys, *T*_max_ = time to maximum plasma concentration, *t*_1/2_ = half-life, CYP = cytochrome P450, TIA = transient ischemic attack, AF = atrial fibrillation, ACS = acute coronary syndrome, FXIa = activated factor XI, TKR = total knee replacement therapy.

In healthy humans, the peak plasma concentration of asundexian was detected at 1 (5–25 mg solution) or 2–5 h (25–150 mg tablet) following oral administration, which corresponded to its rapid onset, and the plasma half-life was about 14–17 h (Table 1).^76^ High-calorie diets slightly reduced its absorption. A reduction in FXIa activity and a prolongation of aPTT persisted over 24 h following asundexian intake.^76^ This pharmacokinetic and pharmacodynamic data supports a once-daily dose of asundexian in humans. There was no increase in bleeding time in all of the tested doses of asundexian.^76^ In vitro, asundexian was proposed to produce mild-to-moderate induction of cytochrome P450 (CYP) 3A4. However, it did not affect the systemic exposure of CYP3A4 substrate (i.e., midazolam) in humans, suggesting no significant impact of asundexian on CYP3A4 activity.^77^

Recently, a phase 2 study of asundexian demonstrated lower rates of bleeding compared to apixaban in patients with atrial fibrillation (PACIFIC-AF trial),^78^ and the evaluation of its efficacy in phase 3 study is ongoing (OCEANIC-AF trial, NCT05643573). In an arterial thrombosis setting, a phase 2 study of once-daily oral asundexian (10, 20, or 50 mg) in combination with dual-antiplatelet therapy for 6–12 months has been performed in 1601 patients with recent acute myocardial infarction (PACIFIC-AMI trial).^79^ The results showed that asundexian appeared to lower ischemic events without an increased risk of bleeding.^79^ However, there is currently no phase 3 investigation of asundexian in patients after acute myocardial infarction.

A phase 2 trial of asundexian has also been undertaken in 1808 patients, mainly after minor noncardioembolic ischemic stroke (National Institutes of Health Stroke Scale [NIHSS] score ≤ 7) (PACIFIC-Stroke trial).^80^ An approximate 2–4% of patients after moderate stroke (NIHSS score 8–15) were also included. All patients had already received single- or dual-antiplatelet therapy.^80^ In this study, once-daily asundexian (10, 20, or 50 mg) was orally administered within 48 h following acute symptom onset. The follow-up period for the combined use of asundexian and antiplatelet therapy was 6–12 months. Although the overall outcomes revealed that asundexian did not reduce ischemic events (the composite of covert brain infarction or ischemic stroke), posthoc analyses demonstrated a significant decrease in the occurrence of TIA with asundexian 20 and 50 mg relative to placebo. In addition, asundexian did not increase the risk of bleeding (the composite of major or clinically relevant nonmajor bleeding). At present, a phase 3 study investigating the efficacy and safety of asundexian in patients after ischemic stroke is ongoing (OCEANIC-STROKE trial, NCT05686070).

## Milvexian

Similar to asundexian, milvexian (JNJ-70033093, BMS-986177) is an orally active small molecule, which specifically and reversibly inhibits the active site of FXIa in a concentration-dependent manner.^90^ Its anticoagulant activity was supported by a potent prolongation of aPTT in human and rabbit plasma without affecting the PT and in vitro aggregation of rabbit platelets induced by adenosine diphosphate (ADP), collagen, and arachidonic acid.^90^ In rabbits, the intravenous administration of milvexian showed a dose-dependent improvement of carotid blood flow and a reduction in thrombus weight following a model of electrically mediated carotid arterial thrombosis (ECAT) with no alteration in bleeding time.^90^

In humans, the maximal plasma concentration of milvexian was observed at 3–4 h after oral intake (Table 1), which allowed rapid onset of FXIa inhibition.^91^ The plasma half-life of milvexian was approximately 8–14 h.^91^ In addition, the aPTT was prolonged over 12 h following 20 or 70 mg of milvexian, supporting twice-daily dosing.^91^ Higher doses (200 or 500 mg) appeared to prolong the aPTT for over 24 h.^91^ The half-life of milvexian was slightly longer (∼18 h) in patients with moderate (eGFR from ≥30 to ≤59 mL/min/1.73 m^2^) and severe (eGFR < 30 mL/min/1.73 m^2^) renal impairment.^92^ Milvexian is a substrate for CYP3A4 (Table 1) and P-glycoprotein (P-gp).^93,94^ Milvexian exposure was moderately increased following concomitant use with multiple doses of itraconazole (a strong CYP3A4 and P-gp inhibitor) but was slightly increased following multiple doses of diltiazem (a moderate CYP3A4 inhibitor).^93^ Notably, a substantial decrease in milvexian exposure was observed after coadministration with multiple doses of rifampin, a potent CYP3A4 and P-gp inducer.^94^ Dosage adjustment is not required in patients with mild-to-moderate hepatic impairment.^95^

Following the report of its efficacy and safety outcomes for VTE prevention in patients undergoing total knee arthroplasty (AXIOMATIC-TKR trial),^96^ a phase 2 secondary stroke prevention trial of milvexian (AXIOMATIC-SPP trial, NCT03766581) was performed in 2366 patients with minor ischemic stroke (NIHSS score ≤ 7) or high-risk TIA (ABCD2 score ≥ 6) with evidence of arterial atherosclerosis. Milvexian was orally administered within 48 h following symptom onset and continued for 90 days. In addition, all patients received dual-antiplatelet therapy (100 mg of aspirin plus 75 mg of clopidogrel) daily for the first 21 days followed by 100 mg of aspirin daily on days 22–90.^13,97^ Although it was not statistically significant, the results revealed that 50 and 100 mg of milvexian twice daily appeared to lower the rate of the primary end point (a composite of ischemic stroke or covert brain infarction detected by magnetic resonance imaging at 90 days). In addition, an approximate 30% relative risk reduction in symptomatic ischemic stroke was observed following treatment with 25–100 mg of milvexian twice daily relative to the placebo. Milvexian was well tolerated with no fatal bleeding or increase in intracranial hemorrhage. The incidence of major bleeding (mainly gastrointestinal bleeds) was moderately increased in patients who took ≥50 mg milvexian twice daily.^13^ Due to these potential benefits, a phase 3 clinical study of milvexian in ∼15 000 patients after an acute ischemic stroke or high-risk TIA is ongoing (LIBREXIASTROKE trial, NCT05702034). Moreover, milvexian is currently undergoing phase 3 trials in patients with atrial fibrillation (LIBREXIA-AF trial, NCT05757869) or in patients after a recent acute coronary syndrome in combination with single- or dual-antiplatelet therapy (LIBREXIA-ACS trial, NCT05754957).

## Other Small Molecule FXIa Inhibitors under Investigation

### SHR2285

SHR2285 was developed as an orally active FXIa inhibitor,^81,98^ but its structure and preclinical data has not been reported in the literature so far. After a single oral administration of SHR2285 (50–400 mg) in healthy individuals, the peak plasma concentration was observed at 3–4 h and the plasma half-life was approximately 8–16 h (Table 1), suggesting twice-daily dosing.^81^ Its active metabolite, SHR164471, had a comparable plasma half-life (10–15 h) to the parent compound. A reduction in FXI activity and a prolongation of aPTT were maintained over 12 h following SHR2285 intake, returning to baseline at 24–48 h.^81^ The reported adverse events of SHR2285 were an increase in conjugated bilirubin and alkaline phosphatase, occult blood positive, and a decrease in neutrophil and white blood cell counts, all of which were self-recovered. There were no serious or life-threatening adverse events.^81^ Moreover, the pharmacokinetic, pharmacodynamic, and safety profiles of SHR2285 (200–300 mg tablet twice daily) were assessed in healthy humans in combination with 100 mg of aspirin plus a P2Y_12_ inhibitor (300 mg clopidogrel loading followed by 75 mg daily or 180 mg ticagrelor loading followed by 90 mg twice daily).^98^ SHR2285 in combination with dual-antiplatelet therapy for 6 days did not alter the time to peak plasma concentration, the half-life, and the FXIa inhibiting activity. In addition, there was no increase in bleeding risk following this triple therapy.^98^ SHR2285 is currently undergoing phase 2 study for the prevention of VTE in patients with total knee arthroplasty (NCT05203705).

### ONO-7684

ONO-7684 (or ONO-5450598) is an imidazole-based selective FXIa inhibitor, which competitively and reversibly binds to FXIa.^82−84^ Intravenous infusion of ONO-7684 prolonged aPTT and decreased thrombus weight in monkeys with the arteriovenous shunt model of thrombosis. In addition, oral administration of ONO-7684 did not alter bleeding time following a nail-cut bleeding model in monkeys, whereas rivaroxaban significantly increased the bleeding time.^82−84^ The oral bioavailability of ONO-7684 varied between species, i.e., 59% in rats, 81% in monkeys, and 88% in dogs.^82−84^

A phase 1 study in healthy humans demonstrated that the peak plasma concentration of ONO-7684 was detected at 2.5–4 h after oral dosing (20–300 mg tablets) (Table 1), which corresponded to its rapid onset of FXIa inhibition.^83^ The plasma half-life of ONO-7684 was 16–20 (fasted) or 22–28 h (fed), allowing once-daily dosing. In addition, a reduction in FXI activity and a prolongation of aPTT were maintained over 24 h following ONO-7684 intake.^83^ Notably, ONO-7684 was well tolerated with no increase in bleeding risk.^83^ To date, there has been no information on proposed clinical trials of ONO-7684 in patients with or at risk of thrombosis.

### BMS-962212

BMS-962212 is a tetrahydroisoquinoline injectable FXIa inhibitor.^85,99^ This small molecule selectively and reversibly inhibits FXIa.^99^ In rabbits with the arteriovenous shunt model of thrombosis, intravenous administration of BMS-962212 significantly reduced thrombus weight and prolonged aPTT without affecting PT.^99^ In addition, BMS-962212 alone or in combination with aspirin did not increase the bleeding time in a rabbit model of cuticle bleeding.^99^

Following a single 2 h intravenous infusion of BMS-962212 (rate of infusion 1.5–25 mg/h) in healthy subjects, the peak plasma concentration was detected within 1–2 h and the half-life was 2–5 h (Table 1).^85^ The maximal effects on the inhibition of FXI activity and prolongation of aPTT were also observed within 1–2 h of BMS-962212 infusion and then approached the baseline at 4–12 h.^85^ Moreover, in a continuous 5-day infusion study (1–20 mg/h), the plasma half-life of BMS-962212 was slightly longer (6–8 h) but the onset and offset of action were comparable to the single-infusion study.^85^ Adverse events following BMS-962212 administration were mild, including infusion site reactions, nausea, headache, upper respiratory tract infection, flatulence, and ecchymosis.^85^ Due to its parenteral route of administration, rapid onset, and relatively short duration of action, BMS-962212 might play a role in acute thrombotic settings. However, there is no current evidence reporting the efficacy and safety of BMS-962212 in patients with or at risk of thrombosis.

### BMS-724296

BMS-724296 is a reversible and selective FXIa inhibitor (Table 1).^86^ A single preclinical study on the antithrombotic activity of BMS-724296 has been reported so far. BMS-724296, administered intravenously, significantly reduced thrombus weight, increased carotid blood flow, and prolonged aPTT in a cynomolgus monkey model of ECAT in a similar manner to apixaban and dabigatran.^86^ However, BMS-724296 did not affect PT and kidney bleeding time, indicating a low bleeding risk relative to an increase in PT and bleeding time following administration of apixaban and dabigatran.^86^

### BMS-654457

BMS-654457 is a tetrahydroquinoline derivative, which reversibly and selectively inhibits FXIa (Table 1).^87^ Similar to BMS-724296, the antithrombotic activity of this small molecule has been demonstrated in a preclinical study. Intravenous administration of BMS-654457 significantly increased carotid blood flow in a dose-dependent manner following a rabbit model of ECAT.^87^ The increased carotid blood flow was correlated with a prolonged aPTT. BMS-654457 did not affect platelet aggregation upon stimulation with ADP, arachidonic acid, or collagen. In addition, BMS-654457 did not alter the bleeding time in a rabbit cuticle bleeding model, suggesting a minor impact on hemostasis.^87^

### BMS-262084

BMS-262084 is 4-carboxy-2-azetidinone-containing FXIa inhibitor.^87,89^ Unlike other FXIa inhibitors, this small molecule irreversibly inhibits FXIa with a half maximal inhibitory concentration (IC_50_) of 2.8 nM.^88^ BMS-262084 increased aPTT without affecting the PT in vitro and ex vivo. In rats, the intravenous administration of BMS-262084 has been shown to reduce thrombus weight and increase carotid blood flow in a model of FeCl_3_-induced carotid artery injury (Table 1).^88^ In addition, BMS-262084 significantly decreased thrombus weight following a rat model of FeCl_3_-induced injury of vena cava (i.e., venous thrombosis) but not in a model of TF infusion.^88^ Although it acts as an irreversible inhibitor, BMS-262084 did not increase the bleeding time when assessed using three models, including cuticle incision, template incision of the renal cortex, or puncture of small mesenteric blood vessels, indicating its low bleeding propensity.^88^

Consistent with data in rats, the intravenous administration of BMS-262084 increased carotid blood flow in a rabbit model of ECAT in a dose-dependent manner, which correlated with a prolonged aPTT.^89^ Moreover, BMS-262084 dose-dependently decreased thrombus weight in rabbits with an arteriovenous shunt model of thrombosis or prosthetic device-induced thrombosis in the vena cava.^89^ BMS-262084 did not inhibit platelet aggregation induced by ADP or collagen. Notably, a high dose of BMS-262084 slightly, but significantly, increased cuticle bleeding time.^89^ At present, there have been no studies reporting the safety and efficacy of BMS-262084 in humans.

## Conclusions

The present evidence clearly suggests that FXIa is a promising target of novel anticoagulants given that it strongly promotes thrombus growth but is less likely to impact hemostasis. With recent advances in the development of FXI-targeting agents, small molecule FXIa inhibitors provide several advantages. First, these small molecules produce a rapid onset of action. Second, many FXIa inhibitors are orally active, which is convenient for the patients. Third, they demonstrate a lower bleeding risk than currently available DOACs, including FXa inhibitors and direct thrombin inhibitor. Fourth, laboratory monitoring might not be required during the use of FXIa inhibitors. Finally, a relatively short half-life of FXIa inhibitors allows rapid recovery in the event of drug-related toxicity or overdose. Therefore, orally active small molecule FXIa inhibitors might represent an interesting new class of effective and safe DOACs. Recently, the role of dual-pathway inhibition (aspirin plus low-dose rivaroxaban) in reducing adverse cardiovascular outcomes has been demonstrated in patients with chronic coronary syndrome or peripheral arterial disease who had a high risk of recurrent ischemia.^100^ However, major bleeding was increased with this drug regimen.^100^ In a phase 2 study in patients after acute myocardial infarction, a combination of asundexian with dual-antiplatelet therapy appeared to reduce ischemic events without increasing the risk of bleeding, indicating that FXIa inhibitors might be an interesting option for dual-pathway inhibition in coronary artery disease. During minor ischemic stroke or high-risk TIA, short-term (21–90 days) dual-antiplatelet therapy using aspirin and clopidogrel is recommended.^101,102^ Recent evidence in patients after acute minor ischemic stroke or high-risk TIA showed no increased bleeding risk from asundexian or milvexian in combination with single- and/or dual-antiplatelet therapy, suggesting a potential indication of FXIa inhibitors for dual-pathway inhibition in cerebrovascular atherothrombosis. The outcomes of ongoing phase 3 trials will provide more apparent directions for the role of asundexian and milvexian in arterial thrombosis. Other FXIa inhibitors require further clinical studies.
